# Investigating CFTR and KCa3.1 Protein/Protein Interactions

**DOI:** 10.1371/journal.pone.0153665

**Published:** 2016-04-19

**Authors:** Hélène Klein, Asmahan Abu-Arish, Nguyen Thu Ngan Trinh, Yishan Luo, Paul W. Wiseman, John W. Hanrahan, Emmanuelle Brochiero, Rémy Sauvé

**Affiliations:** 1 Département de Physiologie moléculaire et intégrative and Membrane Protein Research Group, Université de Montréal, Montréal, QC, Canada, H3C 3J7; 2 Department of Physiology, McGill University, Montréal, QC, Canada, H3G 1Y6; 3 Centre de recherche du Centre hospitalier de l’Université de Montréal (CRCHUM), Tour Viger, 900 rue Saint-Denis, Montréal, QC, Canada, H2X 0A9; 4 Department of Medicine, Université de Montréal, Montréal, Canada, H3C 3J7; 5 Chemistry & Physics, McGill University, Montreal, QC, Canada, H3G 1Y6; Sackler Medical School, Tel Aviv University, ISRAEL

## Abstract

In epithelia, Cl^-^ channels play a prominent role in fluid and electrolyte transport. Of particular importance is the cAMP-dependent cystic fibrosis transmembrane conductance regulator Cl^-^ channel (CFTR) with mutations of the CFTR encoding gene causing cystic fibrosis. The bulk transepithelial transport of Cl^-^ ions and electrolytes needs however to be coupled to an increase in K^+^ conductance in order to recycle K^+^ and maintain an electrical driving force for anion exit across the apical membrane. In several epithelia, this K^+^ efflux is ensured by K^+^ channels, including KCa3.1, which is expressed at both the apical and basolateral membranes. We show here for the first time that CFTR and KCa3.1 can physically interact. We first performed a two-hybrid screen to identify which KCa3.1 cytosolic domains might mediate an interaction with CFTR. Our results showed that both the N-terminal fragment M1-M40 of KCa3.1 and part of the KCa3.1 calmodulin binding domain (residues L345-A400) interact with the NBD2 segment (G1237-Y1420) and C- region of CFTR (residues T1387-L1480), respectively. An association of CFTR and F508del-CFTR with KCa3.1 was further confirmed in co-immunoprecipitation experiments demonstrating the formation of immunoprecipitable CFTR/KCa3.1 complexes in CFBE cells. Co-expression of KCa3.1 and CFTR in HEK cells did not impact CFTR expression at the cell surface, and KCa3.1 trafficking appeared independent of CFTR stimulation. Finally, evidence is presented through cross-correlation spectroscopy measurements that KCa3.1 and CFTR colocalize at the plasma membrane and that KCa3.1 channels tend to aggregate consequent to an enhanced interaction with CFTR channels at the plasma membrane following an increase in intracellular Ca^2+^ concentration. Altogether, these results suggest 1) that the physical interaction KCa3.1/CFTR can occur early during the biogenesis of both proteins and 2) that KCa3.1 and CFTR form a dynamic complex, the formation of which depends on internal Ca^2+^.

## Introduction

The CFTR protein is a plasma membrane cAMP-regulated Cl^−^ channel which mediates transepithelial salt and fluid transport in the airways, intestine, kidney, vas deferens, and sweat duct [1]. Defective CFTR activity causes cystic fibrosis (CF), a disease characterized by an impaired Cl^-^ secretion and unbalanced Na^+^∕Cl^-^transport that leads to airway surface liquid (ASL) volume depletion and defective mucociliary clearance [2–4]. Besides CFTR, other Cl^-^ channels have also been identified at the apical membrane of many epithelial cells. Much attention has recently been given to TMEM16A, a member of the TMEM16 gene family which codes for a Cl^-^ channel activated by internal Ca^2+^ (CaCC). Studies using TMEM16A^-/-^ mice have confirmed that the loss of TMEM16A strongly affects Ca^2+^ -dependent Cl^-^ transport in airways, colonic epithelia, acinar cells from pancreas and submandibular glands [5;6].

The bulk transepithelial transport of Cl^-^ ions needs however to be coupled to an increase in K^+^ conductance to recycle K^+^ brought into the cell by the Na^+^/K^+^ pump and to maintain an electrical driving force for anion exit across the apical membrane. In the airways, two main K^+^ channels, namely, KvLQT1 and KCa3.1 channels have been associated with the control of Cl^−^ secretion. For instance, chromanol 293B- or clofilium-sensitive short-circuit currents have been documented in normal and CF nasal cells, tracheal cells, Calu-3 cells, normal NuLi, and CF CuFi bronchial cell lines, and in alveolar cell monolayers as well [7]. Moreover, pharmacological inhibition of KvLQT1 and KCa3.1 channels was found to strongly reduce Cl^−^ transport in nasal, tracheal, and bronchial cells [7]. Conversely, Ussing chamber measurements have demonstrated that the KCa3.1 channel potentiator 1-EBIO could enhance short-circuit currents in non-CF human airway cell monolayers [8;9]. This induced current was abolished by charybdotoxin (CTX) [9] or Tram-34, a specific inhibitor of KCa3.1 [10]. Notably, evidence has recently been presented indicating that 1-EBIO could potentiate the cAMP-induced Cl^-^ secretion in tissues from CF patients with residual CFTR function (F508del/Y161C or F508del/V233D for instance [11]). These results would thus be in line with a model where KCa3.1 channels provide the necessary driving force to sustain a CFTR-dependent Cl^-^ efflux in epithelial cells [12]. In addition, data have demonstrated that cAMP-activated Ca^2+^ signaling is required in human airway serous cells for CFTR mediated fluid secretion, in support of a mechanism in which cAMP activates CFTR to serve as the secretory Cl^-^ channel, while inducing an internal Ca^2+^ rise that would cause in parallel KCa3.1 channel activation [13].

Until recently, K^+^ channels involved in the control of anion secretion in airway epithelial cells were thought to be exclusively located at the basolateral membrane [7;14]. Increasing evidence suggests however that such K^+^ channels may also be expressed at the apical side of airway epithelial cells. For instance Moser *et al*. [15] have detected KvLQT1 channel expression and chromanol 293B-sensitive KvLQT1 currents at the apical membrane of Calu-3 monolayers using immunocytochemistry and electrophysiological methods. Ussing chamber experiments on non-CF human airway cell monolayers have also demonstrated that basolateral as well as apical 1-EBIO can increase the short-circuit current, an effect which is abolished by Tram-34 [10]. These observations were interpreted as evidence for the presence of functional KCa3.1 channels at both the apical and basolateral membranes of human airway epithelial cells. Similar results were obtained in the human bronchial epithelial cell line 16HBE14o- [16]. Of interest, patch clamp evidence has also been presented for a colocalization of the KCa1.1 (Maxi K) channel and CFTR at the apical membrane of exocrine gland acinus [17]. There was in this particular case no clear indication of a functional coupling between KCa1.1 and CFTR.

An important consequence of KCa3.1 channels being present at the apical membrane of Cl^-^ secreting epithelia is the possibility of both functional and physical protein/protein interactions between CFTR and KCa3.1. Interactions of CFTR channels with ion channels, transporters, receptors, or other signaling molecules have already been documented [18;19]. While the exact mechanism that underlies altered cAMP regulation of ENaC in CF airways is still under debate, there is clear evidence that CFTR and ENaC co-immunoprecipitate and undergo fluorescence resonance energy transfer. CFTR has also been shown to interact with the inwardly rectifying ATP-sensitive renal K^+^ channel ROMK2 [20]. Although CFTR-ROMK2 interactions did not translate into modified gating properties of ROMK2, the binding of CFTR was found to modulate ROMK2 sensitivity to the sulfonylurea compound glibenclamide [20;21]. Protein/protein interactions involving CFTR could thus constitute an important mechanism to modulate fluid transport in epithelial cells. In contrast to CFTR, very few ion channels have been reported to directly interact with KCa3.1. In salivary glands KCa3.1 was found to inhibit Maxi KCa (KCa1.1) channel activity in a voltage independent manner [22]. It was proposed that KCa3.1 channels may be located sufficiently close to KCa1.1 so that activation of KCa3.1 channels causes their N-termini to insert through the cytoplasmic side portals of KCa1.1 resulting in inhibition of ion permeation through the KCa1.1 pore [22]. Co-immunoprecipitation and confocal imaging experiences also demonstrated a close spatiotemporal interaction between KCa3.1 and the Ca^2+^ permeable Orai1 channel in human lung mast cells [23]. It was suggested that the KCa3.1 activity was in this case controlled by Ca^2+^ microdomains, and that KCa3.1 and Orai1 form part of a macromolecular complex organized by PDZ-containing scaffolding proteins [23]. In addition to ion channels, KCa3.1 was documented to form an immunoprecipitable complex with the β-1 integrin in ATII cells [24]. The latter observation suggests the possibility of protein/protein interactions involving KCa3.1 and membrane spanning proteins.

Based on functional evidence suggesting apical expression of KCa3.1 channels in Cl^-^ secreting epithelial cells, a study was undertaken to evaluate possible protein/protein interactions between KCa3.1 and CFTR. We show for the first time that wt-CFTR or F508del-CFTR and KCa3.1 channels can directly interact in normal or F508del-CFTR airway cell lines. We also show that KCa3.1∕CFTR co-expression does not affect CFTR membrane trafficking and cell surface expression. Moreover, we demonstrate that the colocalization of tagged EGFP-CFTR and KCa3.1-dsRed channels, as revealed by image cross-correlation spectroscopy (ICCS) measurements [25], is concomitant with the elevation of intracellular Ca^2+^ concentration and the appearance of KCa3.1/CFTR channel clusters.

## Materials and Methods

### Bacteriophage two-hybrid analysis

A two-hybrid interaction assay was performed as previously reported [10] using the BacterioMatch II Two-Hybrid System (Stratagene) which is suitable for the characterization of protein/protein interactions between pairs of proteins cloned in the pBT (bait) and the pTRG (target) vectors. KCa3.1 or CFTR cytosolic domains were subcloned in the pBT vector (bait) as fusion constructs with the bacteriophage λ repressor protein containing the amino-terminal DNA-binding domain and the carboxyl terminal dimerization domain. The target protein was fused to the N-terminal domain of the α-subunit of RNA polymerase in the pTRG vector. When the bait and target interact, they recruit and stabilize the binding of RNA polymerase at the promoter and activate the transcription of the HIS3 reporter gene. All the transformants grew on a nonselective screening medium, which does not contain 3-amino-1,2,4-triazole (3-AT), a competitive inhibitor of His3 enzyme, but contained chloramphenicol and tetracycline, to indicate that the transformants carried the bait and prey plasmids. Positive signals were verified by using the aadA gene, which confers streptomycin resistance as a secondary reporter (+++). The N-terminal domain of α-RNA-polymerase (pTRG-empty) was used as negative control. The CFTR/NT (M1-P355), CFTR/NBD1 (P335-T665), CFTR/NBD2 (G1237-Y1420) and CFTR/CT (T1387-L1480) segments were chosen to encompass the CFTR N-terminus (M1-R80), the nucleotide binding domain 1 NBD1 (N423-G646), the nucleotide binding domain 2 NBD2 (M1210-P1443) and the CFTR C-terminus region (K1420-L1480), respectively. Because previous studies have demonstrated that AMPK-α1 binds to the COOH-terminal tail of CFTR at residues1420–1457 [26], the AMPK-α subunit was used as a positive control for CFTR/CT domain binding interactions. Similarly, binding of the casein kinase-2 (CK2) on CFTR NBD1 was used as control to confirm interactions involving the CFTR NBD1 [27]. As for KCa3.1, five cytosolic regions were considered in our experiments namely; the N-terminal domain (M1-M40), the S2-S3 linker (A81-R103), the S4-S5 linker (R170-R206) and the C-terminus (N300-A400 or L345-A400) region. The specificity of the interactions was verified by one-on-one two-hybrid analysis and the reporter strain was cotransformed using each purified target plasmid paired with the recombinant pBT plasmid.

### Co-immunoprecipitation of KCa3.1 and CFTR channels from CFBE-wt and CFBE-ΔF508 cells

Immunoprecipitation was accomplished using the immunocomplex-capture technique. Briefly, lysis of CFBE-wt and CFBE-ΔF508 [28] cells was performed in 150 mM NaCl, 50 mM Tris-HCl, pH 7.5, 1% Nonidet P40, and 0.5% sodium deoxycholate buffer from the protein A immunoprecipitation kit (Roche Applied Science) following the manufacturer’s instructions [10]. After protein quantification using the Bradford method, 1–2 mg soluble lysate was precleared with 50μl of 50% protein A-agarose suspension. For immunoprecipitation of endogenous KCa3.1 or CFTR proteins, precleared soluble lysates were incubated for 1h with a rabbit anti-KCa3.1 antibody (1:100, Alomone Labs, Jerusalem, Israel) or with a mouse anti-CFTR antibody (Ab596, 1:100, provided by Dr. J. Riordan through Cystic Fibrosis Foundation Therapeutics, Inc.). The immunocomplexes were precipitated by incubation with 50μl of 50% protein A-agarose suspension overnight at 4°C. After washing, proteins bound to beads were collected by centrifugation and eluted. The immunoprecipitated proteins were then resolved on a 7.5% SDS-PAGE gel and revealed by Western blot analysis. The membranes were incubated with either mouse anti-CFTR antibody (Ab596, 1:1000) or with rabbit anti-KCa3.1 antibody (1:300, Alomone) for 18 h.

### Biotinylated KCa3.1 and CFTR pulldown

Biotinylation and pulldown experiments were done as previously described [29]. Briefly, confluent T-Rex HEK cells expressing wt-CFTR and transiently transfected with HA-tagged KCa3.1 were rinsed three times with glycine quenching buffer and solubilized in RIPA buffer for 15 min on ice. The resulting lysate was centrifuged at 32,000g for 15 min at 4°C, and total cellular protein content was determined using the BCA (bicinchoninic acid) protein assay (Pierce Biotechnology). The supernatant was incubated with streptavidin beads for 30 min at 4°C (10–50 *μ*g of protein/*μ*l of beads pre-equilibrated with RIPA buffer). After centrifugation, the supernatant was removed, the beads were washed five times with RIPA buffer, electrophoresed on SDS/PAGE, and transferred to PVDF membranes. Membranes were incubated with TBS containing an anti-CFTR antibody (mAb 450, 1:5000) and antibodies against the Na^+^/K^+^-ATPase *α*-subunit (a5, 1:200; a gift from Dr R. W. Mercer, Washington University, St. Louis, MO, U.S.A.) and HA tag (Sigma H9658) respectively. Blots were washed and incubated with secondary antibodies conjugated to HRP (horseradish peroxidase; used at 1:1000). Immunoreactive bands were visualized by ECL (enhanced chemiluminescence; Amersham Biosciences) and analyzed by densitometry using ImageJ software (National Institutes of Health).

### Cell culture, infection and transfection

CFBE-wt and CFBE-ΔF508 cell lines: The parental CFBE41o- cell line was developed from CF bronchial cells (F508del/F508del) by Dr. D. Gruenert and colleagues [30] and subsequently transduced with wild-type or F508del-CFTR using the TranzVector lentivirus system [31]. The transduced CFBE41o- lines were generously provided by Dr. J. P. Clancy (University of Cincinnati) and cultured as described previously [32]. For co-immonuprecipitation experiments, wt-CFTR (CFBE-wt) or F508del-CFTR (CFBE-ΔF508) CFBE cells were cultured in EMEM (Invitrogen) supplemented with 10% fetal bovine serum (FBS), 2% L-glutamine, and 1% penicillin-streptomycin with 5% CO_2_ at 37°C, in Petri dishes for 8 days, before protein lysis and co-immunoprecipitation experiments. For CFBE cell infection/transfection with EGFP-CFTR and KCa3.1-dsRed, cells were plated on glass coverslips. At 60% confluency, they were transduced using an adenoviral vector directing the expression of EGFP-CFTR (Penn Vector Core, Philadelphia PA) at a multiplicity of infection of 100 plaque forming units/cell in OptiMEM medium supplemented with 100 nM vitamin D3 (Sigma, St. Louis MO). After 1.5 days exposure, cells were washed and kept in fresh OptiMEM for one day, then transfected with KCa3.1-dsRed plasmid DNA at a concentration of 0.5 μg/million cells using an Amaxa Nucleofector system (Lonza, Allendale NJ). The cells were then seeded in glass-bottom Fluoro-Dishes (World Precision Instruments Inc., Sarasota FL) in CFBE medium lacking penicillin-streptomycin. After 18h, cells were washed with fresh CFBE medium lacking penicillin-streptomycin and left to recover for 12 hours. For CFTR expression, CFBE cells were kept in OptiMEM at 29°C overnight before experiments and imaged as individual live cells under these conditions.

For biotinylated KCa3.1 and CFTR pulldown experiments, a stable HEK cell line with tetracycline-inducible CFTR was generated using the Flip-In T-Rex^™^-293 (Invitrogen) cell line according to manufacturer's instructions. Briefly, the host cell genome contained integrated plasmids pFRT/lacZeo and pcDNA6/TR to yield a Flp Recombination Target (FRT) site and Tet repressor under control of the human CMV promoter, respectively. wt-CFTR cDNA was cloned between the Not1 and Apa1 sites in the expression plasmid pcDNA5/FRT/TO, which also contained the FRT site linked to the hygromycin resistance gene for Flp recombinase-mediated integration. pcDNA5/FRT/TO/CFTR was co-transfected with plasmid pOG44 directing the expression of Flp recombinase and clones with stable integration at the FRT site were selected using 200 μg/ml hygromycin B. Expression of wt-CFTR was induced by adding 1 μg/ml tetracycline for 24 h.

### CPA (cyclopiazonic acid) treatment

To induce Ca^2+^ influx, cells were first pretreated with 10 μM cyclopiazonic acid (CPA) in Ca^2+^-free solution for 15 minutes to deplete the ER Ca^2+^ stores and activate store-operated Ca^2+^ entry channels, and then exposed to a solution containing 2 mM Ca^2+^ (supplemented with CPA). Cells were imaged 15 min after the onset of Ca^2+^ influx. Experiments were performed at 29°C in 5% CO_2_ humidified chamber as mentioned below.

### Confocal microscopy and imaging conditions

Time-series images were collected using the LSM-710 confocal microscopy (Carl Zeiss Canada, Toronto ON) equipped with an Argon ion Multiline laser (488 nm and 514 nm, 25 mW) and 561 nm laser (15 mW). For live cell imaging, 1% laser power was chosen to maximize the signal collected while preventing excessive fluorescence photobleaching, which at the end of the time series, was measured to be < 20% of the initial mean fluorescence intensity. EGFP-CFTR and KCa3.1-dsRed were excited by the 488 nm and the 561 nm laser lines, respectively. All imaging was done using a Plan-Apochromat 63x (NA = 1.40) oil objective and confocal pinhole of 1 Airy unit. The digital gain was set at 900. Experiments were performed at 29°C and 5% CO_2_ in a humidified cell incubator mounted on the microscope stage. Each time-series represented an individual cell and was comprised of 800 images of 256 by 256 pixels area focused on the plasma membrane that was in contact with the Fluorodish. These images were collected at a rate of 6.5 Hz, a pixel size of 0.06 μm and a pixel dwell time of 1 μs. To examine dynamics, 20 to 60 image series from individual cells were analyzed per stimulatory condition for the purpose of highlighting CFTR-KCa3.1 interactions before and after internal Ca^2+^ increase.

### TICCS analysis

Complete descriptions of the spatial and temporal auto- and cross-correlation function analysis involved in image correlation spectroscopy (ICS) are detailed elsewhere [33;34]. Here, we briefly outline temporal image cross-correlation spectroscopy (TICCS) which is one of the variants of the ICS family of techniques. The analysis input is one or multiple image time series collected on a fluorescence confocal microscope from which temporal correlation functions are calculated and fit with appropriate decay models. The general expression that defines the spatiotemporal fluorescence intensity fluctuation auto- and cross-correlation functions is:

$$r_{ab}\left(\varepsilon ,\varphi ,\tau \right)=\frac{<\delta i_{a}\left(x,y,t\right)\delta i_{b}\left(x+\varepsilon ,y+\varphi ,t+\tau \right)>}{{<i_{a}>}_{t}\;{<i_{b}>}_{t+\tau }}$$ (1a)

and for spatial lag variables ε = φ = 0, the correlation function is reduced to a temporal correlation function:

$$r_{ab}\left(0,0,\tau \right)=\frac{<\delta i_{a}\left(x,y,t\right)\delta i_{b}(x,y,t+\tau )>}{<i_{a}{>}_{t}<i_{b}{>}_{t+\tau }}$$ (1b)

where δi_a(b)_(x,y,t) = i_a(b)_(x,y,t)—<i_a(b)_>_t_ is the fluorescence intensity fluctuation in microscope detection channel a(b) at pixel location (x,y) and time t. In eq 1b the numerator indicates the ensemble average spatial correlation over all pixel fluctuations in pairs of images separated by a time lag of τ, while the denominator represents the spatially averaged fluorescence intensity over images at time t and t+τ in the time-series. Eq 1b represents the autocorrelation function of a single detection channel if a = b, and the cross-correlation function between detection channels if a≠b. The autocorrelation function decay reflects the dynamics and average residency time of the fluorescently labeled molecule within the focal spot in the image. If cross-correlation function decay is detectable, it represents the focal spot residency time dynamics of two molecules labeled with different fluorophores that are interacting in a common complex. Non-zero cross-correlation function amplitude is indicative of such an interaction of the proteins CFTR and KCa3.1 in this study. The autocorrelation functions were fit to a model for two protein populations diffusing in a two-dimensional system, defined as follows

$$r\left(0,0,\tau \right)=g_{1}\left(0,0,0\right){\left(1+\frac{\tau }{\tau_{d1}}\right)}^{-1}+g_{2}\left(0,0,0\right){\left(1+\frac{\tau }{\tau_{d2}}\right)}^{-1}+c$$ (2)

while the cross-correlation decay was fit to a two-dimensional diffusion model for one slow population as follows:

$$r\left(0,0,\tau \right)=g\left(0,0,0\right){\left(1+\frac{\tau }{\tau_{d}}\right)}^{-1}+c$$ (3)

where the fitting parameters are g(0,0,0) which is the zero time-lag amplitude, c is an offset to account for incomplete decay of the correlation function and τ_d_ is the characteristic diffusion decay time for the labeled molecule. The g(0,0,0) is inversely proportional to the mean number of independent fluorescent entities (clusters) <n_c_> in a focal spot on average. The fitting parameter τ_d_, combined with the lateral e^-2^ laser beam focus radius ω_0_, allow the calculation of the diffusion coefficient:

$$D=\frac{\omega_{0}^{2}}{4\tau_{d}}$$ (4)

The mean number of clusters in the laser beam focal spot <n_c_> can be converted into a cluster density (CD), which is the number of detected entities per μm^2^ as follows:

$$CD=\frac{<n_{c}>}{\pi \omega_{0}^{2}}$$ (5)

while the ratio of <i> and CD provides a measure of the mean degree of aggregation (DA) of the fluorescent particles in the clusters:

$$DA=\frac{<i>}{CD}$$ (6)

The percent of immobilized proteins in the population can be calculated from the fitting parameters c and g(0,0,0) as follows:

$$Immobile\;\%=\;\frac{c}{g\left(0,0,0\right)+\;c}100$$ (7)

The average number of colocalized fluorescent particles in the focal spot area (interacting particles detected in the two channels a and b) is calculated as follows:

$$<n_{ab}>=\frac{g_{ab}(0,0,0)}{g_{aa}\left(0,0,0\right)g_{bb}(0,0,0)}$$ (8)

from which one can calculate the particle interaction fractions for species imaged in a and b:

$$R_{a}=\frac{<n_{ab}>}{<n_{aa}>}$$ (9)

$$R_{b}=\frac{<n_{ab}>}{<n_{bb}>}$$ (10)

where R_a_ reflects the fraction of molecules detected in channel a that interact with molecules in channel b, and R_b_ is the fraction of molecules detected in channel b that interact with molecules in channel a.

### Statistical analysis

The ICS measured parameters were presented as the mean ± SE for n cells. Data sets obtained with different treatments were compared to the control conditions using the unpaired Student t-test. Differences with p < 0.05 were considered statistically different.

## Results

### Cytosolic domains of CFTR and KCa3.1 interact in vitr*o*

Results of two hybrid screening for CFTR and KCa3.1 interactions are presented in Table 1. We first confirmed already known interactions between the AMPK-α1 C-terminus (R407-P550) and CFTR C-terminus domain (T1387-L1480) as well as between the CK2β protein (M1-R234) and the CFTR NBD1 domain (P355-T665). Similarly, we also confirmed interactions between KCa3.1 C-terminus domains (L345-A400 or N300-A350) and AMPK-γ1 (K100-P331) as we previously reported [10]. In each case, the interactions led to the activation of the reporter gene (+++) as expected. Negative controls with the recombinant pBT + pTRG-empty or pBT-empty failed to activate the reporter gene (---) (data not shown). More importantly, our analysis demonstrated that the KCa3.1 C-terminus bait (N300-A400) can physically interact with the C-terminus of CFTR (T1387-L1480) in bacteria, leading to the activation of the reporter gene (+++, Table 1). We failed however to detect two-hybrid interactions between the CFTR/CT fragment and the KCa3.1 N300-A350 segment, arguing for the L345 to A400 fragment being necessary and sufficient for the CFTR/CT/ KCa3.1 (N300-A400) interaction. Our results also indicate that the interaction involving the KCa3.1 N300-A400 region is specific to CFTR/CT, no significant two-hybrid response was observed with the CFTR/NT, CFTR/NBD1 and CFTR/NBD2 domains. Possible interaction was however detected between the KCa3.1 N- domain (M1-M40) and the CFTR/NBD2 segment, but we failed to detect significant two hybrid responses between CFTR and the KCa3.1 S2-S3 and S4-S5 linker domains (data not shown). The observation of the AMPK-γ1 region extending from K100 to P331 interacting with both the CFTR/CT and KCa3.1 in N-(M1-M40) and C-(L345-A400) termini suggests finally the possible formation of a heterotrimer between these three proteins.

**Table 1:** Positive two-hybrid responses observed using cytosolic CFTR and KCa3.1 domains.

	KCa3.1				AMPKα1	AMPKγ1	CK2β
	Met^1^ Met^40^	Asn^300^Ala^400^	Leu^345^Ala^400^	Asn^300^Ala^350^	Arg^407^Pro^550^	Lys^100^Pro^331^	Met^1^ Arg^234^
**CFTR/CT Thr**^**1387**^ **-Leu**^**1480**^	**---**	**+++**	**+++**	**---**	**+++**	**+++**	n.d.
**CFTR/NT Met**^**1**^ **-Pro**^**355**^	**---**	**---**	n.d.	n.d.	n.d.	n.d.	n.d.
**CFTR/NBD1 Pro**^**355**^**- Thr**^**665**^	**---**	**---**	n.d.	n.d.	n.d.	n.d.	**+++**
**CFTR/NBD2 Gly**^**1237**^**- Lys**^**1420**^	**+++**	**---**	n.d.	n.d.	n.d.	n.d.	n.d.
**AMPK-γ1 Lys**^**100**^**- Pro**^**331**^	**+++**	**+++**	**+++**	**---**	n.d.	n.d.	n.d.

The CFTR/NT (M1-P355), CFTR/NBD1 (P335-T665), CFTR/NBD2 (G1237-Y1420) and CFTR/CT (T1387-L1480) segments were chosen to encompass the CFTR N-terminus (M1-R80), NBD1 domain (N423-G646), NBD2 domain (M1210-P1443) and C-terminus region (K1420-L1480), respectively. The KCa3.1 N-terminus and C-terminus were expressed as the M1-M40 and N300-A400 fragments respectively. Control interactions were also monitored between CFTR and the α1 or γ1 subunit of the AMPK protein, and between CFTR/NBD1-CK2. Positive results are expressed as +++.

### Endogenous CFTR and KCa3.1 proteins interact in native non-CF and CF airway epithelial cells

**Fig 1:**
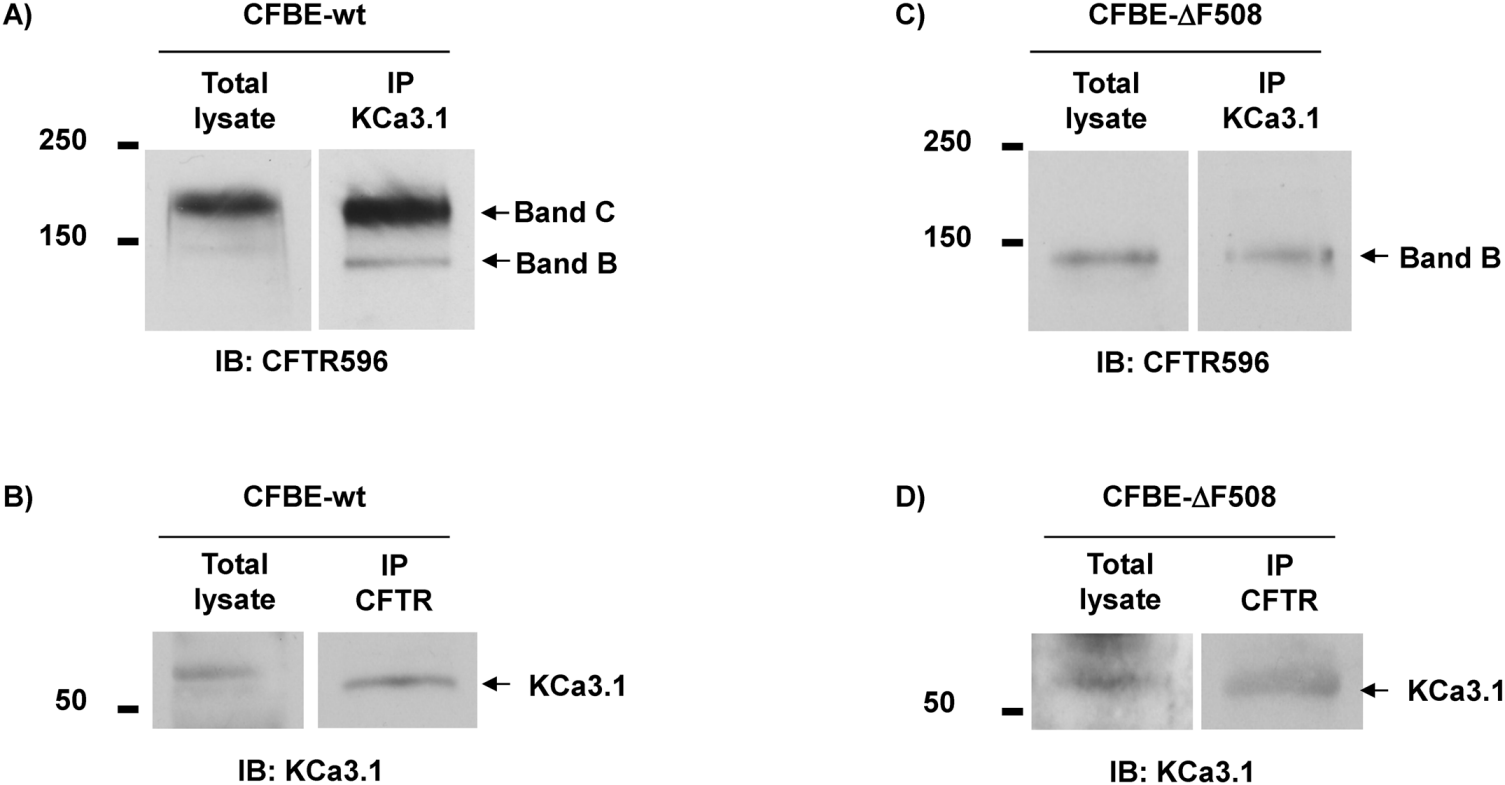
Co-immunoprecipitation of endogenous CFTR and KCa3.1 proteins extracted from CFBE airway cells. Immunoblots showing CFTR and KCa3.1 proteins extracted from CFBE bronchial cells expressing wt-CFTR (A, B) and F508del-CFTR (C, D). Membranes were blotted with anti-CFTR (mAb 596 from CFFT, 1:1000, A, C) and anti-KCa3.1 (Alomone, 1:300, B, D) antibodies. Endogenous expression of CFTR and KCa3.1 proteins in the CFBE-wt and CFBE-ΔF508 cell lysates are presented in lane “Total Lysate”. Immunoprecipitation of endogenous CFTR using anti-CFTR antibody followed by co-immunoprecipitation of KCa3.1 is illustrated in lane IP CFTR (B, D), while immunoprecipitation of endogenous KCa3.1 (using anti-KCa3.1 antibody) followed by co-immunoprecitation of CFTR is shown in lane IP KCa3.1 (A, C). Note that the same lysate and IP samples were used in the upper and lower parts of the membranes, blotted with CFTR and KCa3.1 antibodies, respectively.

Protein/protein interactions between CFTR and KCa3.1 were next evaluated in co-immunoprecipitation experiments in airway epithelial CFBE cells expressing either the wt-CFTR (CFBE-wt) or the F508del-CFTR (CFBE-ΔF508) channels (Fig 1). We first verified that both mature band C and immature band B of wt-CFTR (Fig 1A, lane 1) were detected in CFBE-wt cells, whereas the immature band B of F508del-CFTR only (Fig 1C, lane 1) was present in CFBE-ΔF508 cell lysates, as expected. Immunoblot evidence for endogenous expression of KCa3.1 in both CFBE-wt and CFBE-ΔF508 cells are also presented in Fig 1B and 1D, respectively. More importantly, CFTR C and B bands could be detected by immunoblotting after immnoprecipitation of KCa3.1 (Fig 1A, lane 2), indicating that CFTR and KCa3.1 channels can form immunoprecipitable complexes in CFBE-wt cells. Interestingly, the immature band B of CFTR was also detected after KCa3.1 immunoprecipitation (Fig 1C, lane 2) from CFBE-ΔF508 extracts. Complementary experiments finally confirmed that it was possible to detect the KCa3.1 protein by immunoblot after CFTR protein immunoprecipitation from both CFBE-wt (Fig 1B, lane 2) and CFBE-ΔF508 (Fig 1D, lane 2) extracts, providing clear evidence of a possible physical interaction between CFTR and KCa3.1 in CFBE cells. Since the F508del- CFTR is found almost exclusively in the endoplasmic reticulum, its co-immunoprecipitation with KCa3.1 suggests the physical interaction can occur early during the biogenesis of both proteins.

### Co-expression of KCa3.1and CFTR does not affect CFTR surface expression

**Fig 2:**
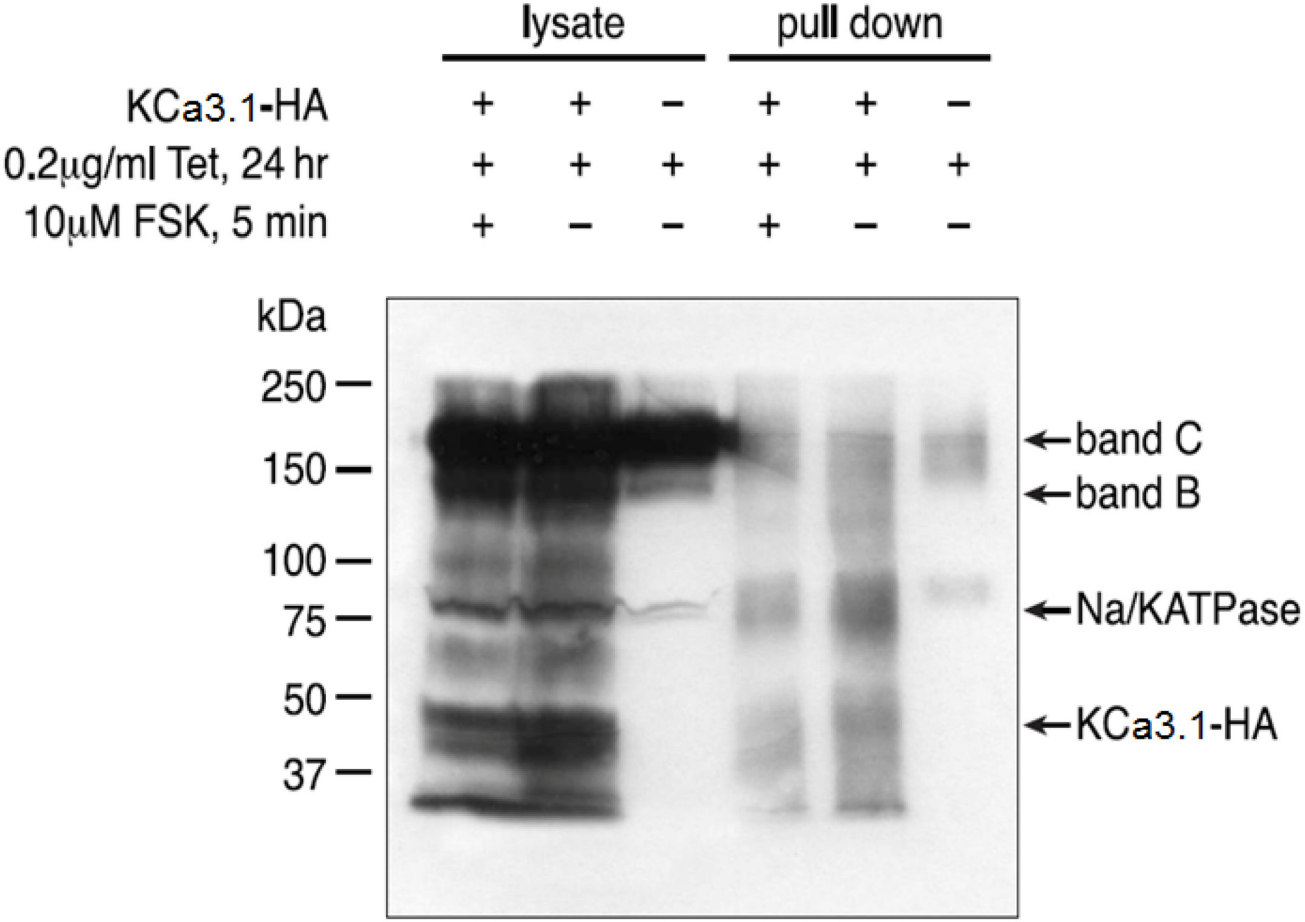
CFTR and KCa3.1 expression in cell lysates and streptavidin pulldowns after cell-surface biotinylation. T-Rex HEK cells expressing WT-CFTR were transfected with HA-tagged KCa3.1 channels and CFTR expression induced by tetracycline (Tet). Lanes 1 and 4 show CFTR, Na^+^/K^+^-ATPase and KCa3.1-HA proteins in the lysate and pulldown (PD) after biotinylation. As controls, lanes 2 and 5 show Na^+^/K^+^-ATPAse and KCa3.1-HA proteins in the lysate and pulldown after biotinylation in absence of forskolin stimulation, and lanes 3 and 6 show CFTR in the lysate and pulldown after biotinylation without forskolin stimulation and KCa3.1 transfection. The molecular mass in kDa is indicated.

Trafficking of CFTR to the plasma membrane of BHK cells has already been investigated using biotinylation and streptavidin pulldown [29]. Because KCa3.1 was found to interact with the CFTR immature form, an identical approach was used to determine if KCa3.1 expression can alter CFTR surface expression. Fig 2 shows a Western blot of T-Rex HEK CFTR-wt cells and streptavidin pulldowns from cells transfected (lanes 1, 2, 4, 5) or not (lanes 3, 6) with KCa3.1-HA tagged channel. In these experiments, CFTR expression was induced by incubating T-Rex HEK CFTR cells in tetracycline during 24h and CFTR was stimulated with 10 μM forskolin (lanes 1 and 4) or not (lanes 2, 3, 5 and 6) before biotinylation and pulldown. CFTR immunoblotting gave a strong and diffuse band of approx. 180kDa as expected after complex glycosylation (band C). This glycoform was enriched by surface biotinylation and pulldown on streptavidin beads (lanes 4, 5, 6). Our results indicated that the surface expression of CFTR did not change whether T-Rex HEK CFTR cells had been cotransfected with HA-KCa3.1 or not. In these experiments, T-Rex HEK CFTR cells non-transfected with KCa3.1 (lanes 3, 6) were used as negative control. Our results also showed that the surface expression of KCa3.1 slightly changed in forskolin CFTR-stimulated cells (lane 4) compared to non-stimulated cells (lane 5). To confirm the effectiveness of our biotinylation and pulldown procedure, we also probed blots for the membrane protein Na^+^/K^+^-ATPase α-subunit, a membrane protein with N-linked glycosylation. The Na^+^/K^+^-ATPase α-subunit was readily detected in T-Rex HEK induced CFTR cells, and its electrophoretic mobility appeared slightly slower in pulldown samples, consistent with an enrichment with the mature form. Altogether, these results show that coexpression of CFTR and KCa3.1 did not significantly change the surface expression of CFTR and that the expression of KCa3.1was not altered by stimulating CFTR.

### TICCS: CFTR interacts with KCa3.1

**Fig 3:**
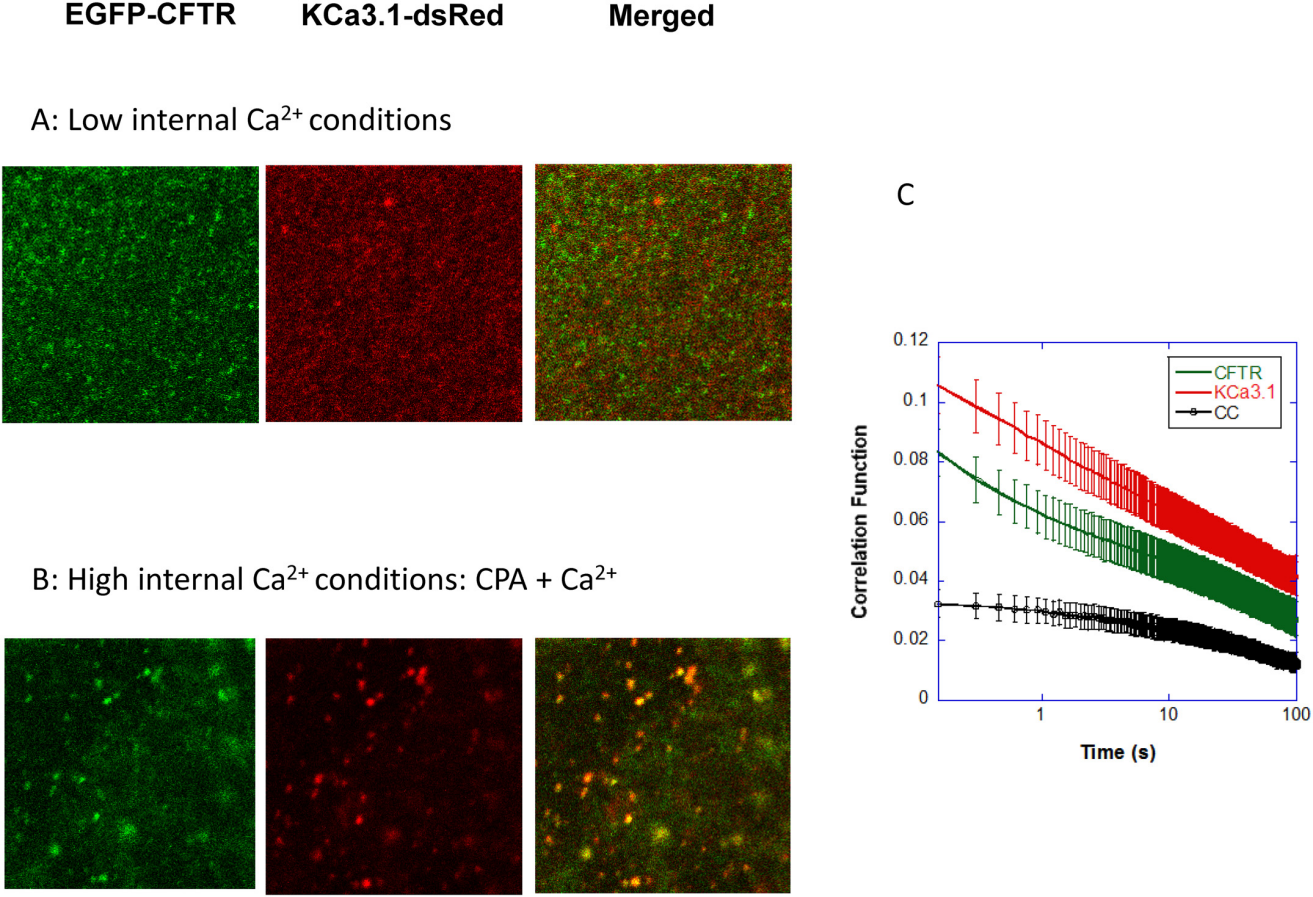
The temporal autocorrelations and the cross-correlation functions of EGFP-CFTR and KCa3.1-dsRed. A: Examples of confocal microscopy images obtained after excitation of EGFP-CFTR and KCa3.1-dsRed at 488 nm and 561 nm respectively, in low Ca^2+^ conditions. Merged figures support colocalization of EGFP-CFTR and KCa3.1-dsRed at the membrane (orange). B: Examples of confocal microscopy images obtained for EGFP-CFTR and KCa3.1-dsRed following CPA pretreatment in zero Ca^2+^ and addition of external Ca^2+^ to initiate Ca^2+^ influx. Merged figures (orange) showed the formation of larger KCa3.1 clusters after internal Ca^2+^ increase. C: Evidence for CFTR/KCa3.1 interactions provided by cross-correlation measurements (see Eq 1b in Materials and Methods section). CFTR-KCa3.1 interactions on the plasma membrane yielded a non-zero cross-correlation function (black) at the slow time scales of the correlation function. This strongly suggests that only the slow populations of CFTR and KCa3.1 interact.

Examples of confocal microscopy images obtained after excitation of EGFP-CFTR and KCa3.1-dsRed in low (A) and high (B) internal Ca^2+^ conditions are presented in Fig 3. In both cases merged figures support colocalization of EGFP-CFTR and KCa3.1-dsRed at the membrane (orange). Additional examples are available within S1 Fig. To assess the average transport dynamics and density of a population of fluorescently labeled KCa3.1 and CFTR channels, fluorescence confocal microscopy image time series were used to calculate the temporal correlation function for CFTR, KCa3.1 and the combination of KCa3.1-CFTR. Fig 3C shows the average auto-correlation functions of CFTR (green) and KCa3.1 (red), and the average cross-correlation function of the interacting population CFTR-KCa3.1 (black) in the plasma membrane of unpolarized, live CFBE cells under resting conditions (control, Ctr). A non-zero cross-correlation function indicates the presence of an interacting population of CFTR and KCa3.1 channels. Both autocorrelation functions exhibited two component decays indicating the presence of two populations with dynamics occurring on different time scales (referred to as fast and slow) for each protein. A model for two diffusing species was used to fit the autocorrelation functions and extract decay times (τ_d_) and amplitudes (g(0,0,0)) and c of the fast, slow and immobile populations for each protein, as indicated in the Materials and Methods section. By contrast, the cross-correlation function exhibited one slow decay component indicating the presence of a single, slowly moving population of CFTR-KCa3.1 interacting in a common complex. This strongly suggests that only the slow populations of CFTR and KCa3.1 interact.

**Fig 4:**
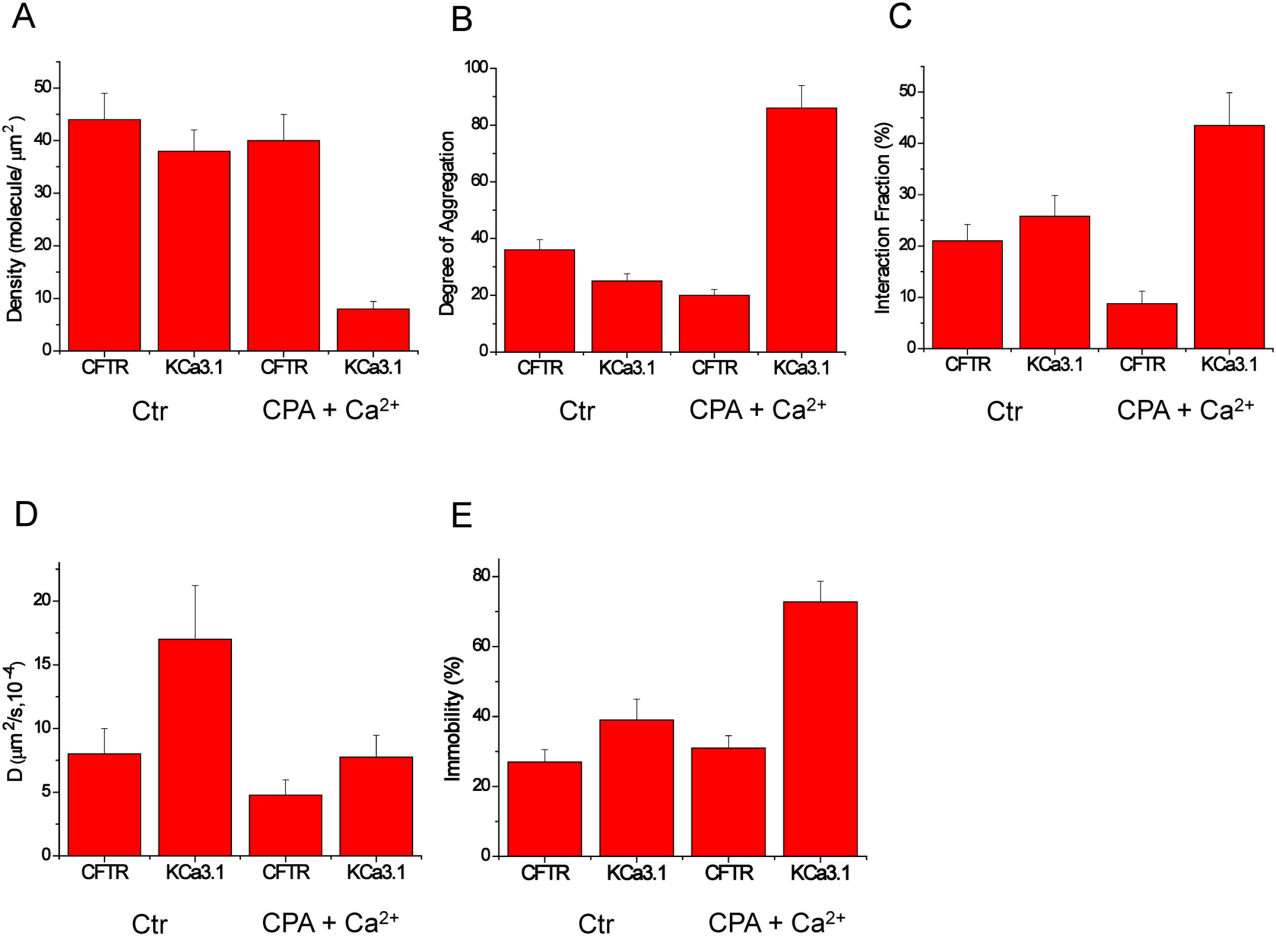
CFTR/KCa3.1 interactions modulated by internal Ca^2+^. A: CFTR and KCa3.1 number densities (molecule/μm^2^) under control and Ca^2+^ influx conditions. KCa3.1 number density dramatically decreases (4-fold) during Ca^2+^ influx induced by pretreatment with CPA (cyclopiazonic acid) followed by exposure to extracellular Ca^2+^, consistent with either internalization of this channel or its clustering. B: CFTR and KCa3.1 degree of aggregation (DA) under control and Ca^2+^ influx conditions. KCa3.1 degree of aggregation increases significantly (4-fold) during Ca^2+^ influx. The 4-fold decrease in number density is accounted for by the 4-fold increase in KCa3.1 cluster size (DA). C: Significant increase in the fraction of KCa3.1 interacting with CFTR in response to a rise in intracellular Ca^2+^ concentration. Protein/protein interactions occurred on a slow time scale (D) and most interactions involved molecules that were immobilized on the plasma membrane (E). Statistical analysis based on n = 62 cells for control experiments and n = 21 cells for measurements in CPA+ Ca^2+^ conditions.

**Fig 5:**
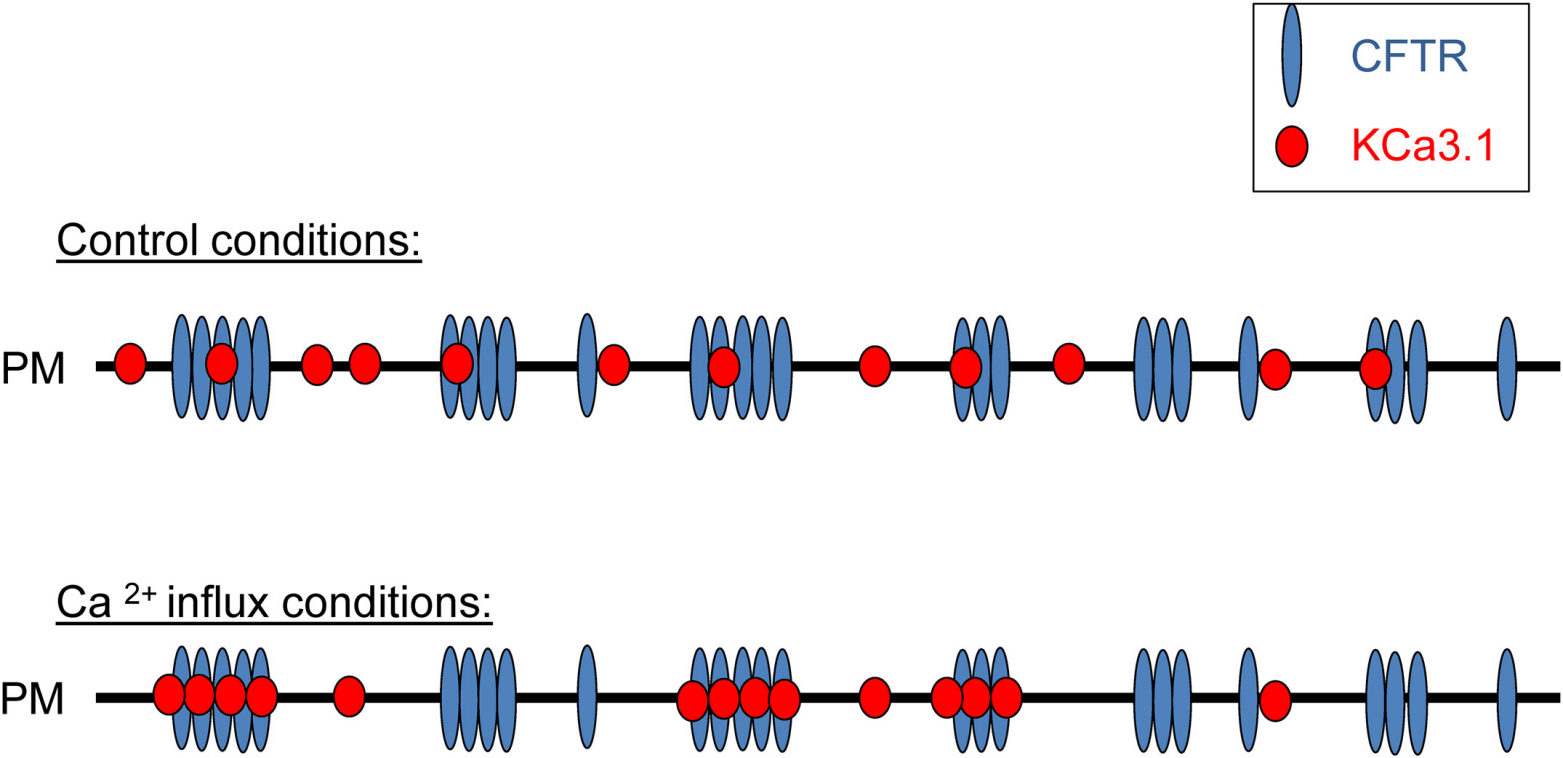
Schematic representation of the effect of internal Ca^2+^ on KCa3.1 interactions and dynamics. Illustration of the increase in CFTR/KCa3.1 clustering in response to an internal Ca^2+^ rise. PM refers to plasma membrane. This scheme accounts for the decrease in KCa3.1 density in the presence of Ca^2+^ as most KCa3.1 channels form aggregates with CFTR.

Comparing the confocal images obtained in low and high Ca^2+^ also revealed a strong effect of internal Ca^2+^ on the CFTR/KCa3.1 interaction pattern. Fig 4A shows in this regard that while the molecular density of CFTR (number of molecules/μm^2^) remained constant before and after Ca^2+^ influx, KCa3.1 density was significantly reduced to < 25% of its original value after Ca^2+^ influx. To determine if this decrease was due to internalization of the K^+^ channels or their clustering with CFTR, the degree of aggregation (DA) was calculated for both proteins (Fig 4B). DA is proportional to the number of fluorescent subunits in each dynamic entity (as defined in the Materials and Methods section); i.e. the higher the DA, the larger the number of fluorescent proteins in the cluster and thus the higher the oligomerization state of the protein complex. While the degree of aggregation of CFTR was not altered by Ca^2+^ influx, a 4-fold increase was seen for the KCa3.1 (Fig 4B). This increase in the size of the KCa3.1 cluster is sufficient to explain the 4-fold decrease in the number density without internalization. We also calculated the interaction fractions of CFTR (R_a_) and KCa3.1 (R_b_) by comparing the amplitudes of the cross-correlation functions with those of the autocorrelation functions. It was concluded that about 20–25% of CFTR and KCa3.1 channels interacted in control low internal Ca^2+^ conditions (Fig 4C; n = 62), compared to 44±5% in high internal Ca^2+^ conditions (n = 21). This indicates that interaction between the two proteins is increased following a rise in intracellular Ca^2+^, with more of the K^+^ channels joining the pre-existing slow population of CFTR molecules. Finally, protein/protein interactions appeared to occur on a slow time scale (Fig 4D) and most interactions involved molecules that were immobilized on the plasma membrane (Fig 4E). The apparent lack of internalization on the time scale of these measurements was also evident from the constant average fluorescence intensity for both proteins at the cell surface before and after Ca^2+^ influx (data not shown). These data are summarized in the schematic model presented in Fig 5, where an increase in KCa3.1 clustering involving CFTR is seen as a result of a rise in internal Ca^2+^.

## Discussion

In this work, we provide the first evidence for a regulated, physical interaction between CFTR and the calcium activated potassium channel of intermediate conductance KCa3.1. This proposal is supported by a two-hybrid screening analysis which showed that the N- and C- regions of KCa3.1 interact with NBD2 and C-terminal regions of CFTR respectively, coupled to results from co-immunoprecipitation experiments indicating that wt-CFTR and F508del-CFTR form an immunoprecipitable complex with KCa3.1 in native non-CF and CF airway epithelial CFBE cells. Notably, our two-hybrid screening procedures also indicated that KCa3.1 and CFTR channels can both interact with AMPK, suggesting a potential indirect coupling of CFTR and KCa3.1 mediated by AMPK. We show in addition that co-expression of KCa3.1 and CFTR does not affect CFTR cell surface expression and that KCa3.1 trafficking is not affected by CFTR stimulation, at least under basal conditions in which the intracellular Ca^2+^ concentration is expected to be low. Finally evidence was obtained through image analysis for the colocalization of tagged EGFP-CFTR and KCa3.1-dsRed channels that would be concomitant to an increase in internal Ca^2+^ concentration. While two proteins may be colocalized by chance, our cross-correlation measurements provided in addition strong evidence for some type of interaction between CFTR and KCa3.1. Using temporal image cross-correlation spectroscopy analysis, we found furthermore that KCa3.1 channels on the plasma membrane aggregate when intracellular Ca^2+^ is elevated, and this promotes interaction with CFTR channels that are already present in uniformly distributed clusters on the plasma membrane as described recently in primary bronchial epithelial cells [35].

### KCa3.1 potassium channel as a part of CFTR regulatory complex

Our two hybrid experiments provide evidence for direct interactions between the KCa3.1 N-terminal region extending from residue M1 to M40 and the CFTR NBD2 region from G1237-K1420, and between the KCa3.1 C-terminal region extending from residue L345 to A400 and the CFTR segment in C-terminus from T1387 to L1480. We showed in a previous work [36] that residues R362 and E363 played a crucial role in channel activation through electrostatic interactions. Importantly these residues were predicted to be highly accessible to solvent, and thus likely to contribute to potential protein/protein interactions. Under these conditions, the binding of CFTR to the L345-A400 region of KCa3.1 could affect the channel activation process without a significant effect on the channel open probability. Furthermore evidence has been presented demonstrating that the Ca^2+^ sensitivity of KCa3.1 is conferred by the binding of Ca^2+^ to calmodulin (CaM), with the CaM C-lobe constitutively bound to a domain in the membrane-proximal region of the KCa3.1 intracellular C-terminus. More importantly the binding of Ca^2+^ to the CaM N-lobe is currently thought to trigger the binding of the CaM N-lobe to a domain in KCa3.1 extending from K360 to K373 which is part of the L345-A400 region predicted in this work to interact with CFTR [36]. The present study raises therefore the possibility of CFTR interfering with the formation of an active KCa3.1/CaM complex or interacting with a 27 amino acid long segment from K373 to A400. In addition, our two-hybrid results indicated that the KCa3.1 N- terminus can interact directly with the NBD2 domain of CFTR. These observations are in line with the documented role of the KCa3.1 N- domain in protein/protein interactions as reported for the KCa3.1/KCa1.1 channel complex, where the first 24 amino acids of the KCa3.1 N-terminus were proposed to act as a KCa1.1 blocker [22]. Evidence supporting KCa3.1/CFTR protein/protein interactions was also obtained in co-immunoprecipitation experiments confirming interactions between KCa3.1 with both CFTR and the F508del-CFTR channel in native airway epithelial cells. The observation that KCa3.1 interacts with the immature form of CFTR would be consistent with KCa3.1 having a chaperone-like function. Immunoblots and measurements of CFTR cell surface expression with or without KCa3.1 co-expression did not however reveal alterations in the amount of mature CFTR or its surface expression. This observation seems to argue against an effect of KCa3.1 on CFTR trafficking, but Ca^2+^ was not buffered during immunoprecipitations and therefore would not have been present at a high enough concentration to promote the interaction. As a result we cannot exclude reciprocal effects on channel trafficking under conditions in which Ca^2+^ levels are elevated, such as during secretagogue stimulation.

We have previously shown a direct interaction between the KCa3.1 C-terminal- region (D360 to A400) which contains coiled-coiled domain (leucine zippers) and the γ1-subunit domain (K100-P331) of the metabolic-sensing Ser/Thr kinase AMPK, with AMPK activation inhibiting KCa3.1 currents in lung epithelial tissues [10]. In the present study we demonstrated an interaction between the N-terminal region of KCa3.1 characterized by the presence of two potential overlapping leucine zippers involved in membrane assembly and trafficking, and the same AMPK-γ1 subunit domain. AMPK could therefore constitute a bridging structure connecting the N and C-terminal domains of adjacent KCa3.1 subunits. In addition, because the AMPK-α1 subunit has been documented to interact with CFTR, our results would be compatible with KCa3.1-CFTR interactions mediated by a complex involving CFTR/AMPK/KCa3.1 with the AMPK-β subunit acting as a scaffold protein, holding the complex altogether. This hypothetical multi-protein complex could be complementary to a direct KCa3.1/CFTR interaction mechanism as suggested by our two-hybrid results.

### KCa3.1-CFTR interactions as a dynamic process

Our TICCS analysis showed that CFTR dynamics and immobility were not altered by Ca^2+^ influx, whereas KCa3.1 responded to a Ca^2+^ rise with a reduced number density, slower dynamics, and increased immobility. In fact, K^+^ channels appeared to aggregate into larger clusters in response to a Ca^2+^ rise, possibly due to their enhanced interactions with CFTR molecules that were already present in uniformly distributed clusters on the plasma membrane (see Figs 3–5). Together, these observations suggest that Ca^2+^ not only regulates the gating of KCa3.1 channels, but also their distribution, dynamics and interaction with other proteins/channels at the plasma membrane. In fact, the results presented in Fig 4 indicating that the conformational changes induced by the binding of Ca^2+^ to the calmodulin-KCa3.1 complex affected both the degree of aggregation and the interaction fraction KCa3.1/CFTR indicate, in our view, a strong functional coupling between the structure of KCa3.1 in C-terminus and the nature of its interactions with CFTR. Such a mechanism could also impact interactions with other transmembrane proteins such as the β-1 integrin and thus contributes to the complementary roles of KCa3.1 and β-1-integrin in the regulation of alveolar repair processes [24]. Because Ca^2+^ binding to the calmodulin (CaM) N-lobe affects the structural organization of the KCa3.1 C-terminus, our TICCS results thus support a clustering process mediated by interactions between KCa3.1 and CFTR C-termini, as revealed by our two-hybrid screen results. Of interest is also the recent observation that a population of CFTR channels showed confinement and slow dynamics that are cholesterol dependent [35]. These lipid conditions are likely to impact on CFTR interaction with other proteins such as KCa3.1.

### Physiological implications

The full physiological implications of the current findings still remain to be established as our TICCS results were obtained using the CFBE cell line. In polarized primary airway epithelial cells, the ratio KCa3.1/CFTR at the apical membrane and the extent of KCa3.1 clustering mediated by the KCa3.1/CFTR interaction remain unknown. However, in polarized epithelial cell monolayers, activation at the apical membrane of a Cl^-^ conductance under conditions of a relatively low paracellular shunt conductance (i.e. in tight epithelia), should lead to a membrane potential at the apical membrane that approximates the equilibrium potential for Cl^-^ ions, which would minimize Cl^-^ efflux [37]. It follows therefore that the concomitant activation of apical K^+^ channels in moderately tight epithelia would constitute a valuable mechanism for insuring that the voltage at the apical membrane remains away from the equilibrium potential of Cl^-^ ions thus maximizing Cl^-^ ions efflux. An equivalent circuit analysis by Cook and Young has concluded in this regard that locating up to 20% of the K^+^ conductance at the apical membrane could increase the secretory rate of Cl^-^ ions due to a less negative transepithelial open-circuit potential [17;38]. Locating more than 20% of the K^+^ conductance at the apical side would however decrease the Cl^-^ ion secretory rate as a result of an apical membrane depolarization coming from an accumulation of K^+^ ions. For leaky epithelial cell monolayers, the control of Cl^-^ efflux at the apical membrane will be however mainly governed by K^+^ channels at the basolateral membrane, with only a small contribution coming from the activation of apical of K^+^ channels. In addition, the possibility of KCa3.1 and CFTR interacting through the metabolic sensing kinase AMPK adds to the complexity of KCa3.1 and CFTR regulation in conditions of low ATP/AMP ratio as encountered during ischemia and airway inflammation.

In conclusion, this study provides evidence for a physical interaction between KCa3.1 and CFTR and points towards a global control of the ion transport properties in Cl^−^ secreting epithelia which is not exclusively mediated by CFTR and ENaC, but which also includes a contribution from KCa3.1.

## Supporting Information

S1 Fig CFTR/KCa3.1 colocalization. Colocalization of CFTR and KCa3.1 on two different cells following increase in internal Ca^2+^.
(TIFF)
