# Supplementary figures and images for: Investigating CFTR and KCa3.1 Protein/Protein Interactions
Source: PLoS One. 2016 Apr 19;11(4):e0153665. doi: 10.1371/journal.pone.0153665 (PMC4836752; doi:10.1371/journal.pone.0153665)

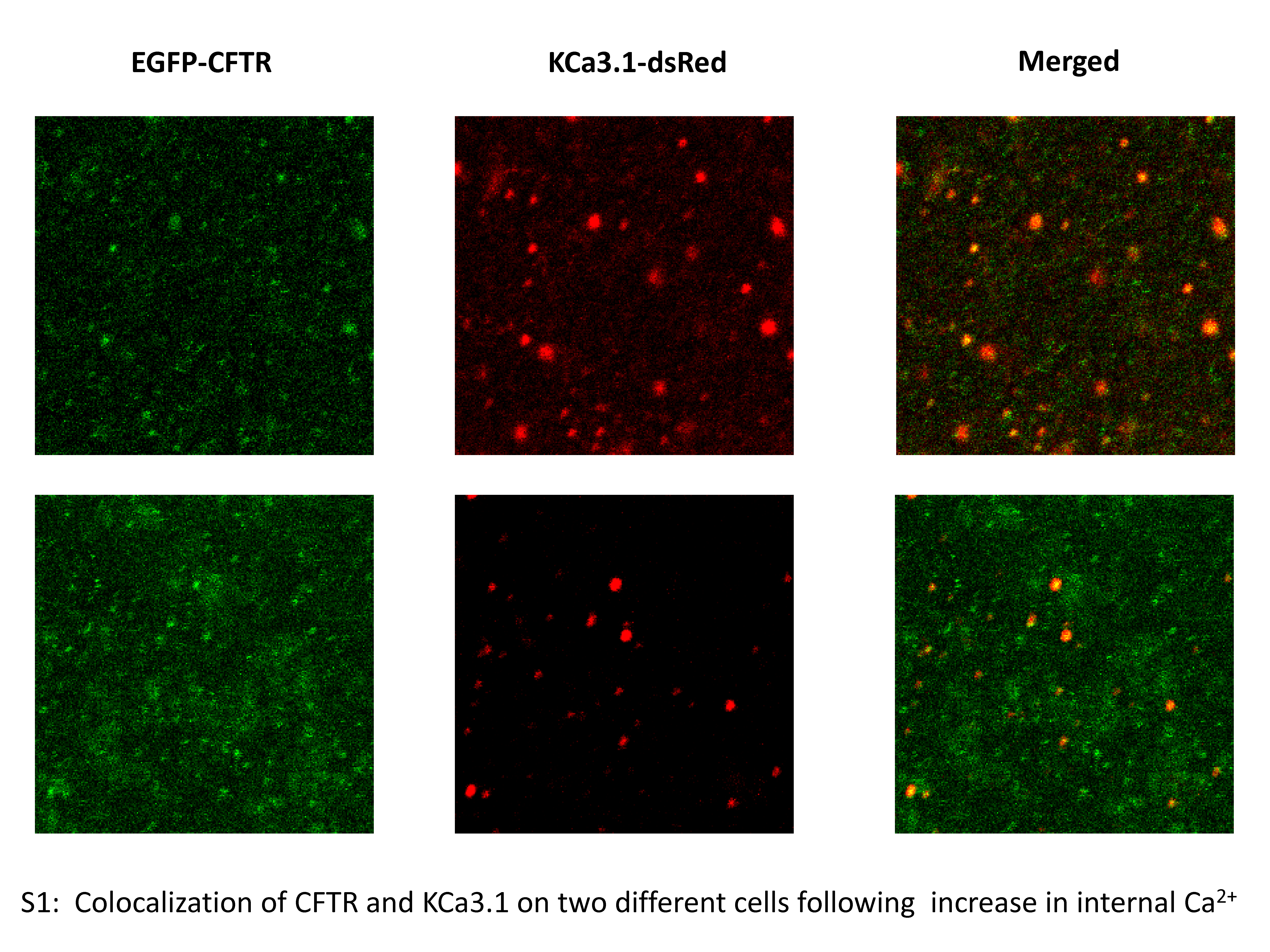

Supplement: S1 Fig — Colocalization of CFTR and KCa3.1 on two different cells following increase in internal Ca2+. (TIFF) [file pone.0153665.s001.tiff]
